# Rapid Development of a Registry to Accelerate COVID-19 Vaccine Clinical Trials

**DOI:** 10.21203/rs.3.rs-4397271/v1

**Published:** 2024-06-10

**Authors:** Neil F. Abernethy, Kylie McCloskey, Meg Trahey, Laurie Rinn, Gail B. Broder, Michele Andrasik, Rebecca Laborde, Daniel McGhan, Scott Spendolini, Senthil Marimuthu, Adam Kanzmeier, Jayson Hanes, James Kublin

**Affiliations:** Biomedical Informatics and Medical Education, University of Washington, 850 Republican St., Seattle, WA 98109,; HIV Vaccine Trials Network (HVTN), COVID-19 Prevention Network (CoVPN), Fred Hutchinson Cancer Center, 1100 Fairview Ave. N., Mail Stop M2-B500, Seattle, WA 98109,; HIV Vaccine Trials Network (HVTN), COVID-19 Prevention Network (CoVPN), Fred Hutchinson Cancer Center, 1100 Fairview Ave. N., Mail Stop M2-B500, Seattle, WA 98109,; HIV Vaccine Trials Network (HVTN), COVID-19 Prevention Network (CoVPN), Fred Hutchinson Cancer Center, Fred Hutch Cancer Center, 1100 Fairview Ave. N., Mail Stop M2-B500, Seattle, WA 98109,; HIV Vaccine Trials Network (HVTN), COVID-19 Prevention Network (CoVPN), Fred Hutchinson Cancer Center, 1100 Fairview Ave. N., Mail Stop M2-B500, Seattle, WA 98109,; HIV Vaccine Trials Network (HVTN), COVID-19 Prevention Network (CoVPN), Fred Hutchinson Cancer Center, 1100 Fairview Ave. N., Mail Stop M2-B500, Seattle, WA 98109,; Oracle Corporation, 2300 Oracle Way, Austin, TX 78741,; Oracle Corporation, 2300 Oracle Way, Austin, TX 78741,; Oracle Corporation, 2300 Oracle Way, Austin, TX 78741,; Oracle Corporation, 2300 Oracle Way, Austin, TX 78741,; Oracle Corporation, 2300 Oracle Way, Austin, TX 78741,; Oracle Corporation, 2300 Oracle Way, Austin, TX 78741,; HIV Vaccine Trials Network (HVTN), COVID-19 Prevention Network (CoVPN), Fred Hutchinson Cancer Center, 1100 Fairview Ave. N., Mail Stop M2-B500, Seattle, WA 98109,

## Abstract

**Background:**

The unprecedented scientific response to the SARS-Cov-2 pandemic in 2020 required the rapid development and activation of extensive clinical trial networks to study vaccines and therapeutics. The COVID-19 Prevention Network (CoVPN) coordinated hundreds of sites conducting phase 2 and 3 clinical trials of vaccines and antibody therapeutics. To facilitate these clinical trials, the CoVPN Volunteer Screening Registry (VSR) was created to collect volunteer information at scale, identify volunteers at risk of COVID-19 who met enrollment criteria, distribute candidates across clinical trial sites, and enable monitoring of volunteering and enrollment progress.

**Methods:**

We developed a secure database to support three primary web-based interfaces: a national volunteer questionnaire intake form, a clinical trial site portal, and an Administrative Portal. The Site Portal supported filters based on volunteer attributes, visual analytics, enrollment status tracking, geographic search, and clinical risk prediction. The Administrative Portal supported oversight and development with pre-specified reports aggregated by geography, trial, and trial site; charts of volunteer rates over time; volunteer risk score calculation; and dynamic, user-defined reports.

**Findings:**

Over 650,000 volunteers joined the VSR, and 1094 users were trained to utilize the system. The VSR played a key role in recruitment for the Moderna, Oxford-AstraZeneca, Janssen, and Novavax vaccine clinical trials, provided support to the Pfizer and Sanofi vaccine and prophylactic antibody clinical trials, and enhanced the diversity of trial participants. Clinical trial sites selected 166,729 volunteer records for follow-up screening, and of these 47·7% represented groups prioritized for increased enrollment. Despite the unprecedented urgency of its development, the system maintained 99·99% uptime.

**Interpretation:**

The success of the VSR demonstrates that information tools can be rapidly yet safely developed through a public-private partnership and integrated into a distributed and accelerated clinical trial setting. We further summarize the requirements, design, and development of the system, and discuss lessons learned for future pandemic preparedness.

## Introduction

The domestic US plan to accelerate SARS-CoV-2 vaccine development aimed to advance multiple stages of clinical trials concurrently. The US government-funded COVID-19 Prevention Network (CoVPN) partnered with the White House and the Department of Health and Human Services (HHS) to evaluate at least two vaccine candidates from each of the three most promising vaccine platforms in tandem.^1^ In addition to aligning and synchronizing clinical trial activities, CoVPN worked to coordinate activities and harmonize data and resources across clinical trials to minimize competitive practices and to increase the value of clinical and immunologic data obtained from these clinical trials. Here, we describe one such coordinated resource: a centralized clinical trial volunteer screening registry (VSR) used to support enrollment in CoVPN clinical trials.

Historically, large clinical trial networks, individual recruiting sites, and clinical research organizations (CROs) have developed and maintained independent, localized volunteer registries. With a national effort spanning several concurrent clinical trials involving over 100,000 participants, a centralized registry was envisioned to prevent redundancy and competition for volunteers. Thus, in May of 2020, we started the development and promotion of a national centralized registry.

The CoVPN VSR was designed to support multi-pronged and concurrent clinical trials, to meet requirements of private vaccine developers, and to interface with public/private clinical research networks. Additional constraints were imposed by the rapid advancement and scaling of vaccine clinical trials in the first months of the COVID-19 pandemic. Furthermore, rapidly evolving scientific evidence influenced the scope and content of screening questions, user queries, and risk estimates needed to support forthcoming clinical trials.

Existing software platforms were initially considered to support a volunteer registry. Several platforms, including customer engagement, electronic data capture (EDC), and clinical trial management systems (CTMS) were evaluated for their capacity to meet functional requirements such as connecting volunteers with clinical trial recruiters and screeners.^2,3^ Candidate systems such as REDCap^4,5^ were customizable, enabled web-based intake, and facilitated volunteer engagement. However, these systems were not designed to scale to handle millions of records and hundreds of simultaneous users. Other commercial software options included patient engagement and recruitment functions but did not meet needs of CoVPN to equitably distribute volunteers, incorporate risk scoring, or perform needed oversight functions. Building a system under an accelerated timeline allowed timely implementation of CoVPN-specific requirements including utilization metrics, scoring functions, and collaboration functions while also scaling to support national and international clinical trials. An existing COVID-19 donation agreement from Oracle Corporation (Oracle) to support HHS-related pandemic efforts enabled us to rapidly collaborate and leverage Oracle technology and development resources to create and deploy the system rapidly.

## Methods

The initial scope, design, and system requirements were determined during the initial wave of the pandemic through collaboration between COVID-19 Prevention Network, HHS/NIH, Oracle, and the overall United States government (USG) operation bridging public and private sector expertise and infrastructure. We solicited additional feedback from vaccine developers, the CDC, and clinical trial sites. The effort encompassed design of a volunteer intake questionnaire, database/user interface (UI) development, user accommodations, and development of risk and targeting algorithms.

### System design

The VSR system requirements encompassed the need for data scale, concurrent users, multiple client interfaces, schema flexibility, adaptability, resilience, compliance, and security. A key requirement was the ability to efficiently store, query, and/retrieve millions of associated records in order to scale to hundreds of thousands of volunteers. Given concurrent recruitment and enrollment around the country, the system was also designed to respond to hundreds of simultaneous push/pull requests. (A summary of system requirements and functions is in Supplemental Table S1.)

Due to the speed of execution, the system was planned and developed in parallel with the volunteer questionnaire and several other aspects of the clinical trial network, even as the clinical trial protocols were still being finalized. To ensure adaptability to changes of the volunteer questionnaire and downstream database use cases, the underlying database architecture was designed to allow for customizable volunteer attributes and value sets.

### User interface and system design

**Figure 1.**
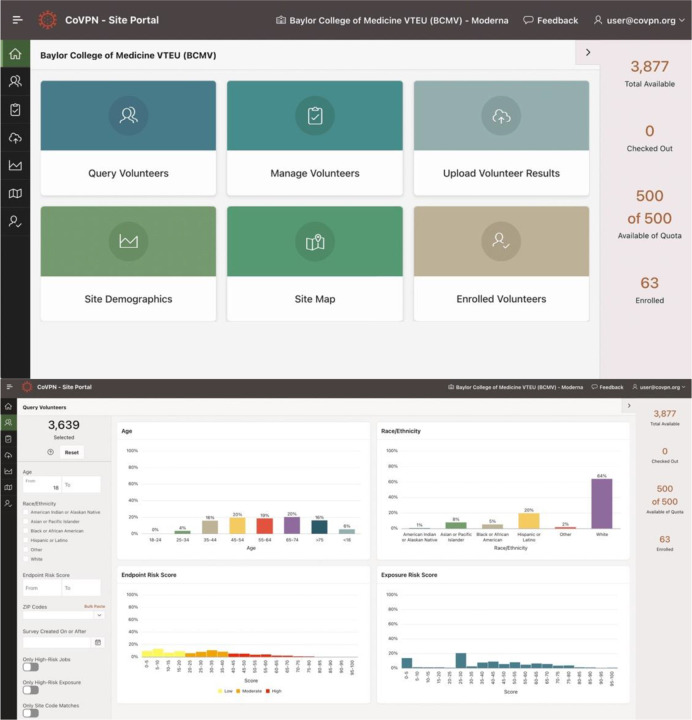

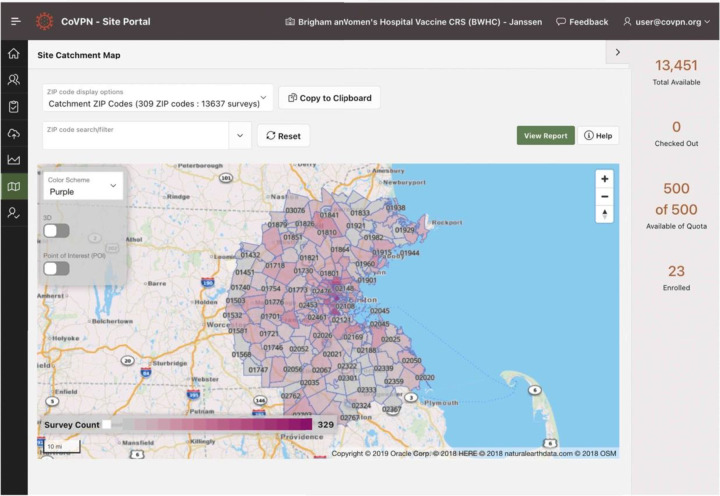
Site Portal. A) Landing page showing six core functions; B) Query volunteers function showing interactive filtering, descriptive statistics of volunteer demographics, and risk scores. C) Interactive map of a site catchment area and volunteer density that enabled selection of volunteers from target regions.

The application UI for clinical trial sites and administrative support were created using rapid prototyping to support recruiters and CoVPN management, respectively. The primary clinical trial site UI consisted of six functions identified in consultation with HVTN and CoVPN leadership, investigators, site Principal Investigators (PIs), and informaticists. The landing page of the Site Portal was designed with simplicity in mind for ease of training, adoption, and use during recruitment (Figure 1).

The underlying relational schema contained 26 tables to support the multi-trial, multi-user nature of the system. The table structure (Suppl. Fig 1) supports various functions: Recording volunteer information (Surveys, Questions, Survey_responses, Question_values, Volunteers, Consent_forms, Risk_scores); Trial information (Trials, Trial_sites, Catchment_areas, Trial_site_zips, Site_users); Survey management (Checkout_sets, Status_codes); and System behavior (Languages, Risk_algorithms, Users, and other event logging). Survey questionnaire responses utilized an Entity-Attribute-Value model to support evolving questions/values from the survey. This approach enabled rapid and relatively seamless changes of the survey without impacting queries that supported system behavior.

Administrative functions evolved more progressively over time as additional management functions and analytics functions were identified. These included monitoring of VSR usage statistics by site, trial, and geographic area; rates and demographics of volunteer accrual; and ad hoc analytics and risk score development.

### Accommodations for users and volunteers

Training materials were developed to help users navigate through the system, understand approaches to screening volunteers, and to share features and requirements of the system. Dozens of virtual training sessions were held by the developer and administrative support teams.

To ensure equitable outreach to a broader population, Spanish language versions of the VSR recruitment website, the VSR questionnaire, and other study materials were provided. A helpline was also established to facilitate registry participation for the elderly or others who faced challenges accessing the registry website.

### Volunteer questionnaire design

Accelerating these clinical trials required rapidly screening volunteers and enrolling hundreds of thousands of participants to meet different study criteria. The VSR questionnaire was designed by a panel of epidemiologists, clinical trial staff, investigators, and community engagement experts to help meet these needs. Registry questions were added to represent demographics and comprehensive clinical, environmental, and behavioral risk factors identified by epidemiologic studies. When possible, questionnaire items were aligned with validated survey instruments or controlled vocabularies, clinical trial enrollment criteria, and emerging literature. The questionnaire (English and Spanish versions) and relevant skip patterns were reviewed for regulatory compliance and approved by Advarra IRB (Pro00044444). The questionnaire and UI were also pilot tested with community members and existing application review panels from different demographic groups to ensure survey comprehensibility and ease of completion. Feedback from these assessments were incorporated into the final questionnaire design.

### Volunteer risk assessment

To optimize the accrual of study endpoints, we calculated risk assessments for individual volunteers. These assessments included published COVID-19 risk models as well as additional clinical and behavioral risk flags and algorithms to estimate the level of exposure, symptomatic disease, hospitalization, intensive care admission, and death. Risk predictions were used to evaluate user queries.

An evidence review was conducted to identify common variables used as inputs for available risk models early in the pandemic. (See Wynants et al. for an earlier review of COVID-19 prediction models.^6^) When possible, these variables such as age, demographics, and other emerging risk factors were included in the volunteer survey.

At the time of initial deployment, given scant published evidence, the VSR supported analytics and volunteer filtering with the COVER-H, COVER-I, and COVER-F risk scores^7^ (estimating risk of hospitalization, ICU admission, and fatality respectively). Support for additional risk models was added during the subsequent four months of VSR deployment. As additional published research on COVID-19 risk accumulated, we synthesized this evidence into additional models to predict the risk of symptomatic disease and SARS-CoV-2 exposure.

The Site Portal provided tools to support volunteer record allotment, management, and status reporting. Signed data use and confidentiality agreements were required for system access, which was further safeguarded by two-factor authentication. A non-identifying volunteer key (distinct from other patient identifiers) was provided to clinical trial sites to enable secure status updates after volunteer contact. Each clinical trial site in the continental United States was limited to screening a fixed number of records within a local catchment area, adjusted for number of competing sites and enrollment targets of the trial. Volunteer records could be “checked out” to prevent competition for volunteers within zip codes targeted by the clinical trials. In September of 2020, queries of the VSR returned volunteer records in order of descending risk to facilitate accrual of study endpoints.

To check out new records, previously accessed records were updated with a status code indicating study enrollment, returning them to the pool of available volunteers, or otherwise removing them from the registry. The system supported a limited set of screening outcomes to simplify use of the system (Contact Not Attempted, Unable to Contact, Reconsider Later, Remove from Registry, Duplicate Record, Contacted/Not Enrolled, and Enrolled). The status codes are shown in Supplemental Table S2. Automated reminders were provided to users if the status of checked out records was not updated within two weeks.

### Role of funding source

Representatives of the funding source (US government) were involved in review of the VSR survey and websites, as well as in site selection, timelines, and protocols for specific CoVPN clinical trials. However, the funding source did not contribute to the analyses or conclusions.

## Results

### System implementation

The CoVPN VSR consisted of distinct user interfaces: the Volunteer Questionnaire; the clinical trial Site Portal for screening volunteers; and an Administrative Portal for oversight of volunteer records, analytics, and site monitoring.

The Volunteer Questionnaire was tested and designed in May-June of 2020 to support varied, projected needs of clinical trials that might hinge on volunteer risk factors, exposures, symptoms, as well as evolving inclusion/exclusion criteria of clinical trials still under active development. This was a challenging task due to the unclear etiology of COVID-19 disease, the contributions of intrinsic versus behavioral risk factors, and the unfolding requirements of each clinical trial. The final components of the questionnaire included *Contact info*, *Demographics*, *Occupation*, *Residence*, *Community interactions*, *COVID-19 risks and testing*, and *Symptoms and health risks*. These records supported volunteer screening and prioritization for multiple clinical trials (the full survey is provided in Supplemental Materials).

The Site Portal was developed as an interface for clinical trial site staff and principal investigators to characterize, query, and access volunteer records for the purpose of volunteer contact. The portal provided six ways of interacting with volunteer records:

- Query volunteers - Selection of tailored subsets of volunteer records
- Manage volunteers - In-browser tools to set volunteer record statuses or return records
- Upload volunteer results - De-identified record status updates via file upload
- Site demographics - Descriptive statistics of volunteers within a site’s catchment area
- Site map - Interactive map and selection tools to identify volunteers within target areas
- Enrolled volunteers - Overview of volunteers marked as enrolled by a site

### Registry Administration

**Figure 2.**
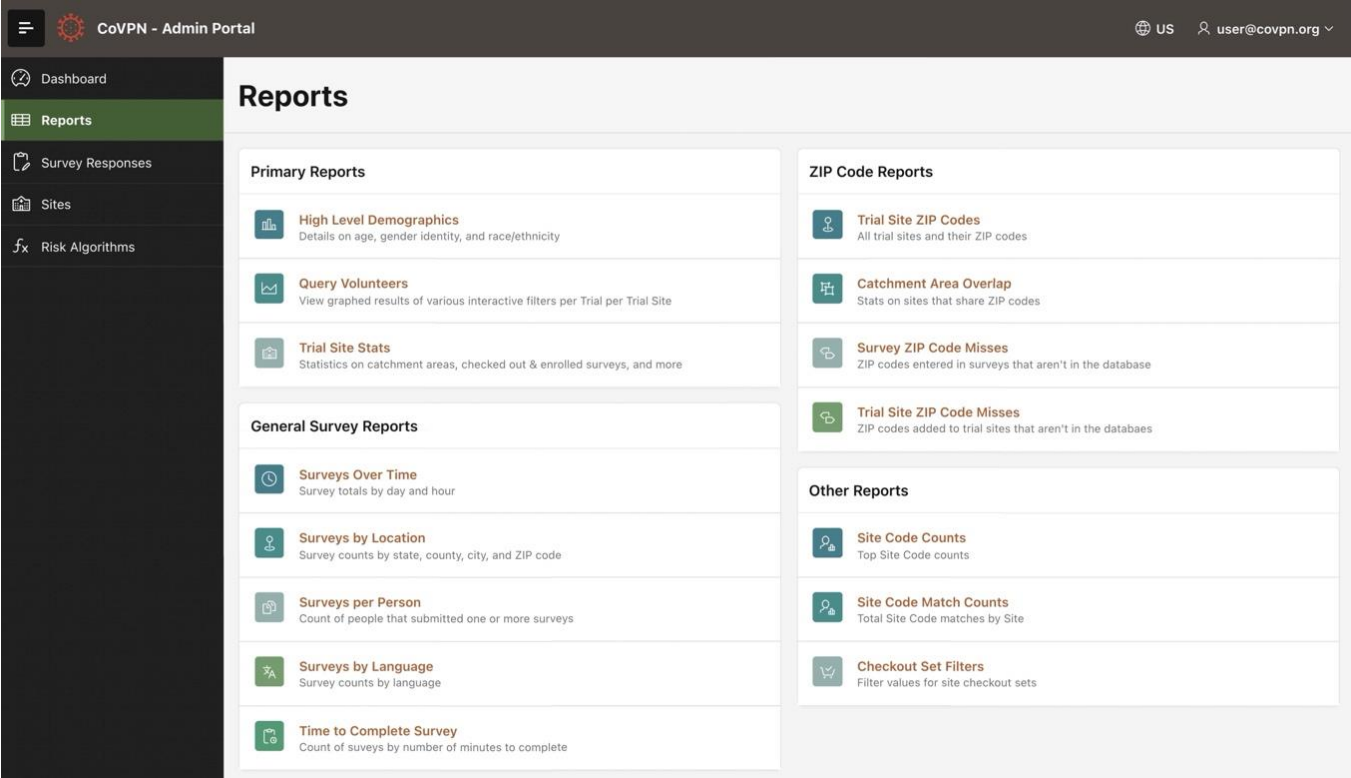
Administrative Portal. A variety of live reports from the database supported periodic monitoring of volunteering, site utilization, and enrollment according to time, geography, or clinical trial.

The Administrative Portal utilized an activity dashboard, and provided standardized reports that facilitated survey response monitoring, site administration, and risk algorithm administration (Figure 2). Several reports were customized to provide survey statistics and site-specific and trial-specific monitoring. The system also supported all functions from the Site Portal at the site level, as well as the ability to pose ad hoc queries, reports, and data visualizations.

### Volunteer and site usage statistics

**Figure 3.**
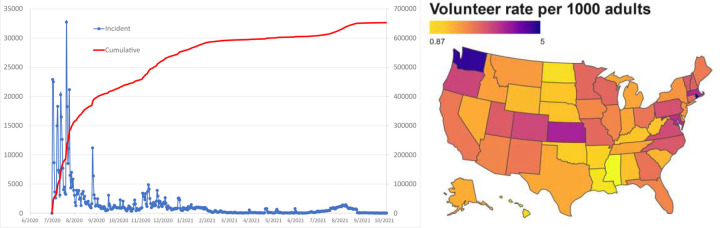
Volunteer registrations over time and geography. Over 300,000 volunteers registered in the system within the first month. The map shows the volunteering rate per 1000 adults in each state (mean = 2·23 / 1000 US adults).

The registry was launched to the public on July 8, 2020, seven weeks after the announcement of federal vaccine development plans.^8^ With 657,750 volunteers in total, the VSR was highly effective; 50% of these volunteers signed up within the first 36 days. Volunteer registrations also increased in concert with public service announcements during August and September (see Figure 3). Participation was also increased using site-specific registration codes, which enabled recruiters to utilize the VSR in concert with volunteer campaigns and events.

The Site Portal was subsequently launched on August 4, 2020. In the US, 455 unique clinical research sites, accessed a total of 168,015 volunteer records for recruitment and enrollment into six phase 3 COVID-19 vaccine clinical trials. These volunteers were drawn from catchment areas that included 22,201 US zip codes. Supplemental Table S3 shows the number of sites involved per clinical trial.

### Evaluation and feedback

The rapid development and deployment of the system limited intensive user-centered design or system evaluations. However, the VSR was publicly credited with advancing the clinical trials. Although the emergency circumstances prevented a formal evaluation, we detail below steps taken to respond to volunteer and user system requirements.

The median time to complete a survey was 5 minutes. The completion rate of the VSR questionnaire was 95·3%.

The VSR survey and Site Portal both allowed submission of comments to the development team. Volunteer feedback on the survey was reviewed weekly to identify any logical errors, omissions, usability concerns, or confusing questions. For a summary of user feedback, see Supplemental Table S4. Site Portal users also requested some functions including support for site- and campaign-specific recruitment codes for volunteers, additional query filters, and the ability to save and retrieve query results. These features were incorporated into updated versions of the system. Thrice-weekly development team meetings and task management software were used to prioritize any bugs or feature requests.

Stage 2 and 3 clinical trials for each vaccine launched on different dates. Therefore, some clinical trials were able to access more diverse and recent volunteers than other clinical trials.

## Discussion

Due to COVID-19 lockdowns and limitations on in-person collaboration, clinical and research teams responding to the pandemic faced unprecedented challenges. In our case, the CoVPN VSR development and administrative teams assembled and collaborated remotely during every phase of the project, encompassing system design, development, documentation, training, technical support, and evaluation. In addition to remote collaboration tools such as Zoom and Microsoft Teams, the team utilized telephone, email, Slack, and web-based office productivity, scheduling, project management, and survey applications to support information sharing and coordination. Team members’ experience with these platforms varied according to institutional licensing for specific technologies, which required compromise as the team formed new work processes.

The network of clinical trial sites engaged for each study was selected through a collaborative process that involved stakeholders from HHS, vaccine developers, CoVPN, and CROs. Because each trial included sites from pre-existing vaccine trial networks as well as newly appointed CRO sites, it was necessary to position the VSR for accessibility and generality instead of specializing for a more uniform user base or the needs of a single developer. To achieve adoption, dozens of training sessions were held with hundreds of recruiters and investigators from different clinical trial sites. Individual sites often employed specialized recruitment procedures or utilized additional digital platforms mandated by vaccine developers or CROs. Future efforts to coordinate clinical trial networks may benefit from establishing plans for secure, standards-based, bidirectional information sharing akin to electronic medical record exchange.

The CoVPN VSR quickly accrued volunteers upon its introduction, reaching over 300,000 within the first month. However, despite ongoing advertising, volunteering rates slowed after the initial few months of availability. Vaccine misinformation and fears were already circulating during this time before the clinical trials were even complete. It is possible that this misinformation affected volunteering rates as well as subsequent recruitment efforts. To combat these efforts, the CoVPN website included information about vaccine science, individual clinical trials, the study process, and frequently asked questions in both English and Spanish.

One chief concern raised from VSR data was the inclusion of potentially sensitive identifying information and health conditions among survey responses. Such data were required to identify volunteers potentially at risk of severe COVID-19 outcomes, to ensure participants could be reached, and to ensure equitable recruitment. The need to collect and store this data might have been obviated by a national health identifier or linkage to electronic health records. Having collected this information, our user interface, training, and data use policy were crafted to promote retention of such data within the VSR system. Collection and use of the data were approved through an Institutional Review Board.

Several alternative COVID-19 clinical trial volunteer registries were developed in the initial months of the pandemic by both commercial and non-profit entities.^9–12^ For example, some registries leveraged electronic health records to identify patients for COVID-19 therapeutic clinical trials, following the clinical cohort-driven model adopted by Clinical and Translational Science Award sites through the Accrual to Clinical Trials initiative (CTSA ACT).^13,14^ This recruitment model was not appropriate for prospective vaccine clinical trials that required healthy volunteers.

Other approaches included vaccine developer-specific, site-specific, region-specific^12,15,16^, or comorbidity-specific^11,17–19^ volunteer registries. Though beneficial for specific recruiting organizations, this approach had the drawback of locking volunteers into one or a small subset of clinical trials for which they might be eligible. Two NIH initiatives provide prior examples of attempts to recruit a broader population of healthy volunteers. ResearchMatch.org is a general volunteer registry to connect eligible volunteers with clinical trials.^20^ In its 12-year history, this CTSA project has accrued 166,289 volunteers. Another NIH program, the Clinical Research Volunteer Program, is designed to support local clinical trials run by the NIH.^21^

### Enabling technologies

The rapid design and implementation of the CoVPN VSR was facilitated by Oracle Application Express (APEX, a low-code application development platform), application programming interfaces, web-based visualization tools, and the Oracle Database (all hosted in Oracle’s Government Cloud). Use of an Entity-Attribute-Value^22^ data definition for questionnaire intake data allowed for mid-flight schema updates which could be quickly passed through to updated user interfaces. This prevented the need for major architecture changes as new COVID-19 risk data and clinical trial priorities became available in the last months of 2020. Web-based project management, video conferencing, chat, file-sharing, and email facilitated rapid collaboration and establishment of new cross-organization teams.

### CoVPN Voluntary Screening Registry impact

Although the full impact of the registry on final clinical trial enrollment is currently unknown, we estimate that it increased enrollment up to 20% in some clinical trials. Given the difficulties commercial CROs had in recruiting diverse participants, adoption of the VSR unquestionably accelerated accrual into clinical trials and increased participation of more diverse populations.^23–25^

In addition to expediting enrollment, the VSR thus served as a crucial recruiting platform to ensure representation of diverse populations in study findings. Moreover, it improved recruitment efficiency by enabling sites to reach volunteers who met preliminary eligibility criteria.

Finally, by achieving an unprecedented geographic coverage in record time, the VSR mitigated the risk of alternative epidemic scenarios or unfavorable clinical trial outcomes, which could have required highly targeted or continuous enrollment.

### Lessons for the next pandemic

Open communication and flexibility were key to rapidly organizing a functional development team and meeting the unique needs of these accelerated clinical trials. The use of specialized teams to focus on system requirements, architecture, rapid iterative design-and-build process, administration, and training allowed the efficient division of labor.

In an emergency situation, competing priorities among stakeholders require compromises in the design and implementation of digital health systems. Public health experts have widely discussed the need for increased surveillance capacity, sustained investments in the public health infrastructure, and investments in data standardization and the health care system to better prepare and respond to pandemics. To support this, such investments must encompass the infrastructure for clinical trials and research IT. Additional facilitators of efficient and coordinated clinical trials include:

- Access to updated data standards, survey instruments, and other artifacts^26^
- Surge capacity among clinical and IT experts
- Harmonized record sharing across EMR, CTMS, laboratory, and other clinical sources
- Flexible funding streams to support partnerships between domain experts and private enterprise

## Conclusion

Through a public-private partnership, remote teamwork, and nimble development, we overcame barriers to collaboration imposed by the pandemic to connect hundreds of thousands of study volunteers to vaccine and antibody clinical trials. Emergency circumstances required balancing different priorities of the US government, sponsors, vaccine and antibody developers, research partners, and the public. Preparedness for future public health emergencies will benefit from continued investments in cross-institutional collaboration tools, data standardization, digital health data linkage, and platforms for public health communication.

## Supplementary Material

1

## Data Availability

Deidentified summary data of volunteer survey completion rates and CoVPN site statistics are available upon email request to the corresponding author upon publication. Data representing enrollment status, geography, demographics will be considered on a case-by-case basis.

## References

[1] Bok K, Sitar S, Graham BS, Mascola JR. Accelerated COVID-19 vaccine development: milestones, lessons, and prospects. Immunity 2021; 54(8): 1636–51. 34348117 10.1016/j.immuni.2021.07.017 PMC8328682

[2] Leroux H, McBride S, Gibson S. On selecting a clinical trial management system for large scale, multi-centre, multi-modal clinical research study. Health Informatics: The Transformative Power of Innovation: IOS Press; 2011: 89–95. 21893916

[3] Choi B, Drozdetski S, Hackett M, Usability comparison of three clinical trial management systems. AMIA Annual Symposium Proceedings; 2005: American Medical Informatics Association; 2005. p. 921. PMC1560441 16779208

[4] Harris PA, Taylor R, Minor BL, The REDCap consortium: Building an international community of software platform partners. Journal of biomedical informatics 2019; 95: 103208. 31078660 10.1016/j.jbi.2019.103208 PMC7254481

[5] Harris PA, Taylor R, Thielke R, Payne J, Gonzalez N, Conde JG. Research electronic data capture (REDCap)—a metadata-driven methodology and workflow process for providing translational research informatics support. Journal of biomedical informatics 2009; 42(2): 377–81. 18929686 10.1016/j.jbi.2008.08.010 PMC2700030

[6] Wynants L, Van Calster B, Collins GS, Prediction models for diagnosis and prognosis of covid-19: systematic review and critical appraisal. BMJ 2020; 369: m1328. 32265220 10.1136/bmj.m1328 PMC7222643

[7] Williams RD, Markus AF, Yang C, Seek COVER: using a disease proxy to rapidly develop and validate a personalized risk calculator for COVID-19 outcomes in an international network. BMC Med Res Methodol 2022; 22(1): 35-. 35094685 10.1186/s12874-022-01505-z PMC8801189

[8] NIH NIoAaID. NIH Launches Clinical Trials Network to Test COVID-19 Vaccines and Other Prevention Tools. NIH Newsroom: News Releases; 2020.

[9] Peeler A, Miller H, Ogungbe O, Centralized registry for COVID-19 research recruitment: Design, development, implementation, and preliminary results. J Clin Transl Sci 2021; 5(1): e152–e. 34462668 10.1017/cts.2021.819 PMC8387691

[10] Romero-Sánchez CM, Díaz-Maroto I, Fernández-Díaz E, Neurologic manifestations in hospitalized patients with COVID-19: The ALBACOVID registry. Neurology 2020; 95(8): e1060–e70. 32482845 10.1212/WNL.0000000000009937 PMC7668545

[11] Rüthrich MM, Giessen-Jung C, Borgmann S, COVID-19 in cancer patients: clinical characteristics and outcome-an analysis of the LEOSS registry. Ann Hematol 2021; 100(2): 383–93. 33159569 10.1007/s00277-020-04328-4 PMC7648543

[12] Salmanton-García J, Stewart FA, Heringer S, VACCELERATE Volunteer Registry: A European study participant database to facilitate clinical trial enrolment. Vaccine 2022; 40(31): 4090–7. 35659449 10.1016/j.vaccine.2022.05.022 PMC9159788

[13] Kost RG, Corregano LM, Rainer T-L, Melendez C, Coller BS. A data-rich recruitment core to support translational clinical research. Clin Transl Sci 2015; 8(2): 91–9. 25381717 10.1111/cts.12240 PMC4405427

[14] Niyibizi N, McIntosh S, Hudson BL, Sipocz A, Paku E, Dykes C. CTSA recruitment resources: An inventory of what CTSA hubs are currently offering. J Clin Transl Sci 2020; 4(6): 529–36. 33948229 10.1017/cts.2020.44 PMC8057486

[15] Hamadah H, Alahmad B, Behbehani M, COVID-19 clinical outcomes and nationality: results from a Nationwide registry in Kuwait. BMC Public Health 2020; 20(1): 1384-. 32912230 10.1186/s12889-020-09490-y PMC7482377

[16] Methi F, Hart RK, Godøy AA, Jørgensen SB, Kacelnik O, Telle KE. Transmission of SARS-CoV-2 into and within immigrant households: nationwide registry study from Norway. J Epidemiol Community Health 2021: jech-2021–217856. 10.1136/jech-2021-217856 34930811

[17] Freeman EE, McMahon DE, Lipoff JB, The spectrum of COVID-19-associated dermatologic manifestations: An international registry of 716 patients from 31 countries. J Am Acad Dermatol 2020; 83(4): 1118–29. 32622888 10.1016/j.jaad.2020.06.1016 PMC7331510

[18] Hearst N. Review 1: “HIV infection and COVID-19 death: population-based cohort analysis of UK primary care data and linked national death registrations within the OpenSAFELY platform”. MIT Press - Journals; 2020. 10.1016/S2352-3018(20)30305-2 PMC7773630 33316211

[19] Tang Y, Li C, Gururangan K. Reviews of “Neurological manifestations associated with COVID-19: a nationwide registry”. MIT Press - Journals; 2021.

[20] Harris PA, Scott KW, Lebo L, Hassan N, Lighter C, Pulley J. ResearchMatch: a national registry to recruit volunteers for clinical research. Academic medicine: journal of the Association of American Medical Colleges 2012; 87(1): 66. 22104055 10.1097/ACM.0b013e31823ab7d2 PMC3688834

[21] Health NIo. Office of Human Subjects Research.(2006). Guidelines for remuneration of research subjects in the intramural research program and registration in the clinical research volunteer program database.

[22] Nadkarni PM, Marenco L, Chen R, Skoufos E, Shepherd G, Miller P. Organization of heterogeneous scientific data using the EAV/CR representation. Journal of the American Medical Informatics Association 1999; 6(6): 478–93. 10579606 10.1136/jamia.1999.0060478 PMC61391

[23] Miller L, Tirrell M. Moderna slows coronavirus vaccine trial enrollment to ensure minority representation, CEO says. CNBC, Health and Science; 2020.

[24] Andrasik MP, Broder GB, Wallace SE, Chaturvedi R, Michael NL, Increasing Black, Indigenous and People of Color participation in clinical trials through community engagement and recruitment goal establishment. PLOS One 2021; 16: e0258858. 34665829 10.1371/journal.pone.0258858 PMC8525736

[25] Jaklevic MC. Researchers Strive to Recruit Hard-Hit Minorities Into COVID-19 Vaccine Trials. JAMA 2020; 324(9): 826–8. 32789501 10.1001/jama.2020.11244

[26] Richesson RL, Nadkarni P. Data standards for clinical research data collection forms: current status and challenges. J Am Med Inform Assoc 2011; 18(3): 341–6. 21486890 10.1136/amiajnl-2011-000107 PMC3078665

