# Supplementary material for: Rapid Development of a Registry to Accelerate COVID-19 Vaccine Clinical Trials
Source: Res Sq. 2024 Jun 10:rs.3.rs-4397271. Preprint. [Version 1] doi: 10.21203/rs.3.rs-4397271/v1 (PMC11213164; doi:10.21203/rs.3.rs-4397271/v1)
Supplement: 1 [file NIHPPrs4397271V1-supplement-1.pdf]

Supplemental Figure 1. CoVPN Registry database schema.

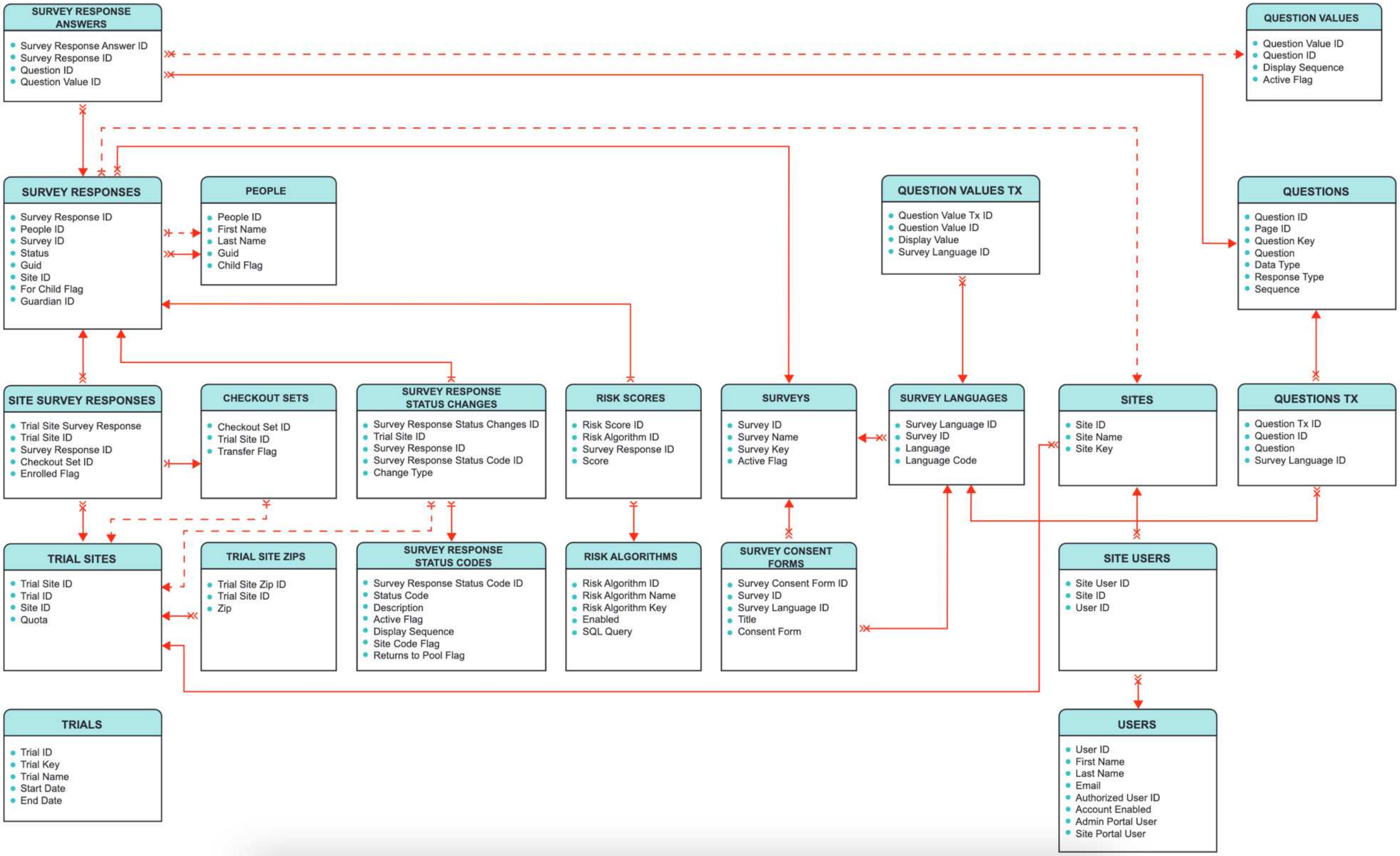

Supplemental Table S1 - Overview of specialized CoVPN Registry requirements and functions

	Functional requirement	Rationale	Enabling function or Implementation
**Volunteer survey**	Unique volunteer & record identifiers	Enable volunteers to update surveys	Many-to-one relationship b/t volunteers and surveys
		Volunteer privacy	Redundant internal and sharable IDs
	Risk score inputs	Leverage existing evidence to identify those with high likelihood of exposure or symptomatic disease	Standardized survey instrument questions and data elements
	Validation rules	Ensure high quality of user-entered data	Browser- and internal database rules and constraints; data validation queries
	High-bandwidth and high-capacity storage and retrieval	Multiple concurrent users of registry survey (volunteers) and portal (sites) to enable rapid screening	Enterprise-grade and cloud-hosted database and web services
**Clinical Trial Site Portal**	View demographics and summary statistics of local volunteer population	Identify sub-populations that meet inclusion criteria and recruiting goals	Interactive charts<br>Choropleth maps
	Filter volunteer records by demographics, risk, and geography	Enroll targeted groups more efficiently	User interfaces for filtering and selecting records based on volunteer details
	Volunteer screening tools	Quickly assess volunteer eligibility and interest and prevent redundant contact	In-browser UI for rapid status code assignment and volunteer follow-up
	Update volunteer statuses	Track enrollment progress; prevent redundant contact	Status codes and functional removal of volunteers from registry search results upon record checkout or volunteer request
	Save and reuse screening criteria	Improve ability to find new volunteers fitting established criteria	User-specific naming, reproduction, and storage of search queries
	Search for volunteers by outreach campaign	Improve follow-up of volunteers most likely to participate while providing credit to recruiters	Site- and campaign-specific recruitment codes stored with volunteer surveys and searchable in UI
**Administrative Portal**	Detailed reports of volunteer statistics	Monitor volunteering rates, populations, and diversity campaign results	Stored administrative queries linked to analytics and visualizations,
	Monitor site and trial record access and accrual progress	Improve site and record utilization; ensure trial enrollment progress and status codes of participants	Real-time report generation with accrual charts and statistics, broken out by targeted metrics including demographic representation
	Upload site information and catchment areas;<br>Restrict access to volunteer information	Scope new sites/users to appropriate volunteer populations	Tables and queries return volunteer records only in site-specific local zip codes
	Add and edit pre-computed volunteer risk scores	Increase speed and efficiency of interactive tools; adapt database to new risk evidence	Score computation prototyping and validity/ security checking prior to deployment

Supplemental Table S2. Volunteer record status codes

	**Meaning**	**Result**
**Code**		
5	Contact Not Attempted: no attempt to email/call was made.	Returned to pool – others can query
10	Unable to Contact: contact attempted but unsuccessful. Used after all contact attempts have been made.	Returned to pool – others can query
20	Contacted - Not Right Now, Consider Me Later: not moving forward now, but interested in this study or another study in the future.	Returned to pool – others can query
30	Contacted - Remove from Registry: not interested in this or any study now or later – do not contact again.	Removed from registry
35	Duplicate Record: More than one survey was completed by the same person. Retain the latest record and return the other(s) with code 35.	Duplicates removed from registry, latest record retained
40	Contacted - Not Enrolled: contacted and may have screened, but ultimately did not enroll. May still be eligible for other studies.	Returned to pool – others can query
50	Enrolled: has been randomized into the study and vaccinated (per definition in the protocol).	Removed from registry

Supplemental Table S3. Number of sites utilizing the registry from each clinical trial.

	Sites participating	Records accessed
**Trial**		
AstraZeneca mAb (PROVENT)	39	838
AstraZeneca mAb (STORM CHASER)	54	7854
AstraZeneca Vaccine	71	59,318
CoVPN 3006 - Transmission Study	53	5117
Janssen	122	18,892
Janssen 2 Dose	2	271
Moderna	91	40165
Novavax	113	30,777
Novavax Adolescent	9	149
Pfizer	19	15
Sanofi Phase 2	9	2612
Sanofi Phase 3	122	721
Total	**	166,729

** Note that several sites participated in more than one non-concurrent trial, so this column does not sum to the total number of sites cited in the paper.

Supplemental Table S4 – Count of Individual User Feedback Items by Category.

	**Count**
**User feedback category**	
Communication and Contact	5
Inclusion, Eligibility, and Language	3
Information Accessibility	11
Location and Logistics	3
Miscellaneous	4
Protocol Questions	7
Survey Clarity and Understanding	22
Symptom Reporting and COVID-19 Awareness	2
Technical Issues	2
User-Friendly Design	7

Feedback on the VSR survey was solicited from diverse users during internal pilot testing. Sixty-six unique items of feedback were grouped into the discrete categories above, reflecting the frequency of questions or concerns raised. This feedback, as well as ongoing feedback from users of the live system, were used to iteratively improve the design and usability of the VSR.
